# Gross’ Edition of Liston’s Elements of Surgery

**Published:** 1842-09

**Authors:** 


GROSS' EDITION OF LISTON'S ELEMENTS OF SURGERY.

This book, which was announced as forthcoming in June last, is now before the public, and has met with unqualified approbation from the eastern press. We have not yet received a copy, but hope to obtain one in time to present to our readers, in the journal for October, such notice of its contents as will enable them to form some idea of the merits of the work. C.

